# Vitamin D as a Potential Therapy for Multiple Sclerosis: Where Are We?

**DOI:** 10.3390/ijms21093102

**Published:** 2020-04-28

**Authors:** Samiksha Wasnik, Isha Sharma, David J. Baylink, Xiaolei Tang

**Affiliations:** 1 Division of Regenerative Medicine, Department of Medicine, Loma Linda University, Loma Linda, CA 92350, USA; 2 Department of Pathology & Medicine, Northwestern University, Feinberg School of Medicine, Chicago, IL 60611, USA; 3 Department of Veterinary Biomedical Sciences, College of Veterinary Medicine, Long Island University, Brookville, NY 11548, USA

**Keywords:** multiple sclerosis, experimental autoimmune encephalomyelitis, vitamin D, 1,25(OH)_2_D, hypercalcemia

## Abstract

Multiple sclerosis (MS) is a chronic demyelinating disease of the central nervous system and is caused by an aberrant immune response to myelin sheath. Disease-modifying medications, which mainly aim to suppress such aberrant immune response, have significantly improved MS treatment. However, the disease severity continues to worsen. In contrast, progressively more data suggest that 1,25-dihydroxyvitamin D or 1,25(OH)_2_D, i.e., the active vitamin D, suppresses the differentiation of potentially pathogenic T cells associated with MS, enhances the differentiation of regulatory T cells that suppress the pathogenic T cells, and promotes remyelination. These novel 1,25(OH)_2_D functions have encouraged investigators to develop vitamin D as a potential therapy for MS. However, because of the hypercalcemia that is associated with high 1,25(OH)_2_D concentrations, supplementation of native vitamin D has been a major focus in clinical trials for the treatment of MS, but such trials have produced mixed data. In this article, we will review current progress in the supplementation of different vitamin D forms for the treatment of experimental autoimmune encephalomyelitis (i.e., an MS animal model) as well as MS. Furthermore, we will review alternative strategies that our laboratory and others are pursuing in an attempt to circumvent the hurdles that are hampering the effective use of vitamin D as a potential therapy for MS.

## 1. Introduction

Multiple sclerosis (MS) is a chronic demyelinating disease of central nervous system (CNS) and afflicts 2.5 million individuals worldwide [1]. The prevalence and incidence are still increasing worldwide. Compelling evidence suggest that the disease is caused by an aberrant immune response to myelin sheath. Such an aberrant immune response damages myelin sheath and causes secondary loss of neurons, eventually leading to disability and even death. So far, disease-modifying medications, which aim to suppress inflammation in CNS, have significantly delayed disease progression, reduced disease severity, and improved life quality among MS patients. However, the disease severity continues to worsen. Therefore, development of novel therapeutic strategies is necessary.

Based on the studies of experimental autoimmune encephalomyelitis (EAE, which is a major universally accepted MS animal model), major primary pathogenic T cells in MS include Th1 and Th17 cells [2,3] and major predominant protective T cells include regulatory T (Treg) cells such as FoxP3^+^ and IL-10^+^ Treg cells. In this regard, data from studies of EAE in our laboratory and others have shown that 1,25-dihydroxyvitamin D or 1,25(OH)_2_D, i.e., the active vitamin D, suppresses the differentiation of Th1 and Th17 cells [4,5] but enhances the differentiation of Treg cells such as FoxP3^+^ and IL-10^+^ Treg cells [6,7,8]. Hence, 1,25(OH)_2_D can theoretically stop the worsening of MS severity by suppressing pathogenic T cells.

In addition, recent data suggest that a major reason for the worsening of MS severity is the failure of remyelination [9]. Further analyses have demonstrated that the remyelination failure is mainly due to a failure in the differentiation of oligodendrocyte progenitor cells (OPCs) into mature oligodendrocytes but not a failure in the migration and proliferation of OPCs because abundant OPCs can be seen at demyelinated lesions in CNS [10,11,12]. In this regard, 1,25(OH)_2_D has been shown to enhance remyelination by promoting the differentiation of OPCs [10,13]. Hence, in addition to potentially stopping the worsening of MS severity by suppressing pathogenic T cells, 1,25(OH)_2_D can theoretically promote recovery from MS by enhancing remyelination.

In summary, the aforementioned previous findings strongly support the notion that vitamin D supplementation can be a potential therapy for MS. In this article, we will review the progress in the study of vitamin D supplementation for the treatment of MS and EAE, hurdles that are hampering the progress of this field, as well as potential strategies that may help to circumvent such hurdles.

## 2. Vitamin D Metabolism

Vitamin D can be obtained from dietary sources. Food has two forms of vitamin D, i.e., vitamin D2 and vitamin D3. Vitamin D2 comes from plants and vitamin D3 comes from animals. However, the most important source of vitamin D in an individual is skin, where vitamin D is synthesized from 7-dehydrocholesterol through UV-B irradiation. In the classical pathway of vitamin D metabolism, the skin-derived vitamin D is first converted to 25-hydroxyvitamin D or 25(OH)D by 25-hydroxylase in liver. 25(OH)D is further metabolized to 1,25(OH)_2_D in kidney by 25-hydroxyvitamin D 1α-hydroxylase (1α-hydroxylase) [14]. 1,25(OH)_2_D executes its biological functions by binding to vitamin D receptor (VDR) and is eventually converted into biologically inactive calcitroic acid by 24-hydroxylase [15].

With respect to 25-hydroxylase, different types are present in different tissues [14]. However, liver is the major tissue that contains 25-hydroxylase. Even in liver, there are also several types of 25-hydroxylase such as CYP2R1 and CYP27A1. CYP2R1 was identified in the microsomal fraction of mouse liver and CYP27A1 was identified in mitochondria. Previous data demonstrated that CYP2R1 knockout and CYP2R1/CYP27A1 double knockout mice only displayed approximately 50% reduction in serum 25(OH)D levels. These data indicate that CYP2R1 is the major but not the only enzyme that converts vitamin D into 25(OH)D and that 25-hydroxylases in tissues other than liver also contribute to serum 25(OH)D levels. Interestingly, the CYP2R1/CYP27A1 double knockout mice still maintained normal serum levels of 1,25(OH)_2_D [16]. Because even a 50% reduction in serum 25(OH)D levels do not affect serum 1,5(OH)_2_D levels, the regulation of 25-hydroxylation should not be a major concern [14].

In contrast to 25-hydroxylase, of which there are several types, CYP27B1 is the only enzyme that has been shown to have 1α-hydroxylase activity [14]. Consistently, humans with genetic mutations in CYP27B1 have pseudovitamin D-deficient rickets due to inadequate 1,25(OH)_2_D production [17,18]. Based on these findings, it can be stated that CYP27B1 has a major role in the production of 1,25(OH)_2_D. In addition, it has been well established that 1α-hydroxylase in kidney is the major if not the sole contributor for the circulating levels of 1,25(OH)_2_D. As a consequence, patients with kidney failure are deficient in circulating 1,25(OH)_2_D. In kidney, 1α-hydroxylase is regulated primarily by three hormones: i.e., parathyroid hormone (PTH), fibroblast growth factor 23 (FGF23), and 1,25(OH)_2_D. PTH stimulates, but FGF23 and 1,25(OH)_2_D inhibits the expression of 1α-hydroxylase [19,20,21]. Besides kidney, in 1980, Turner et al. for the first time reported in vitro synthesis of 1,25(OH)_2_D by calvarial cells [22], suggesting the presence of extra renal 1α-hydroxylase. Now, it is well established that 1α-hydroxylase is expressed in cells of many tissues such as those cells in the immune system [23,24]. Studies of tissue-specific knockout and overexpression of CYP27B1 have substantiated the importance of these extra-renal 1α-hydroxylases. Results of these studies demonstrate that tissue-resident 1α-hydroxylase is indeed critical for the integrity of tissue functions [25,26]. Importantly, there are differences in the regulation of extra renal compared to renal 1α-hydroxylase. This will be discussed further below. Moreover, it appears that regulatory mechanisms of these extra-renal 1α-hydroxylases differ among tissues [14,23]. In summary, the aforementioned studies suggest that tissue-specific regulation of 1α-hydroxylase is necessary for maintaining functional integrity of a tissue.

Similar to CYP27B1 that is the only known 1α-hydroxylase, CYP24A1 is the only known 24-hydroxylase. As mentioned above, 24-hydroxylase degrades 1,25(OH)_2_D into biologically inactive calcitroic acid. In humans, inactivating genetic mutations in CYP24A1 have been identified in children patients with idiopathic infantile hypercalcemia. These patients were highly sensitive to vitamin D supplementation. Upon vitamin D supplementation, these patients presented with hypercalcemia, hypercalciuria, nephrocalcinosis, as well as reduced serum levels of PTH and 24,25(OH)_2_D [27]. These previous data suggest that the major function of CYP24A1 is to prevent the toxic effects of 1,25(OH)_2_D. Since CYP24A1 is expressed in all tissues, it is probable that appropriate regulation of CYP24A1 is necessary for maintaining the optimal levels of 1,25(OH)_2_D in a tissue. From a clinical standpoint, the finding that 1,25(OH)_2_D stimulates the activity of CYP24A1 may explain in part why large doses of 1,25(OH)_2_D are required for a suitable localized effect, such as in the CNS of MS patients (see below).

## 3. Association of Vitamin D Status with MS Incidence and Disease Activity

As early as 1974, the work by Goldberg [28] showed that the incidence of MS was inversely related to the degree of sunlight exposure. Since sunlight exposure is the major source of vitamin D, it was hypothesized that curtailed supply of vitamin D is associated with a high risk of MS. Such hypothesis has driven the investigations on the potential association between vitamin D status and the incidence and disease activity of MS. Currently available data appear to suggest that the levels of serum vitamin D, determined as serum levels of 25(OH)D, affect the risk of MS and also modify the disease activity in MS patients [29]. These previous association studies prompted further investigations that assessed the role of vitamin D in the treatment of MS. In the following sections, we will review the results of ongoing investigation of vitamin D supplementation as a potential therapy for MS.

## 4. Preclinical Studies on the Role of Supplementations of Different Vitamin D Forms in Demyelinating Diseases

### 4.1. Role of 1,25(OH)_2_D Supplementation in the Treatment of EAE

As mentioned above, preclinical studies suggest that 1,25(OH)_2_D has at least three biological functions that can potentially halt the progression and promote the recovery of MS. Such potentially therapeutic functions for MS include 1,25(OH)_2_D’s ability to suppress the differentiation of Th1 and Th17 cells [4,5], enhance the differentiation of Treg cells such as FoxP3^+^ and IL-10^+^ Treg cells [6,7,8], and promote remyelination [10,13]. The next question is whether 1,25(OH)_2_D can effectively execute these functions in animals induced for EAE. In 1991, it was demonstrated that intraperitoneal administration of 0.1 μg but not 0.05 μg 1,25(OH)_2_D every other day beginning three days before EAE induction reduced the incidence and severity of paralytic disease [30]. A subsequent study further showed that 1,25(OH)_2_D at one initial intraperitoneal dose of 300 ng followed by a daily diet of 20 ng, when initiated at disease onset, prevented the progression of paralytic disease. In addition, this therapeutic effect is reversible because removal of the treatment led to a relapse of the disease [31]. Another observation in the above studies was that the preventive and therapeutic doses of 1,25(OH)_2_D were associated with hypercalcemia. The authors discussed that “the hypercalcemia induced by the dosages of 1,25(OH)_2_D required to produce optimal immunosuppression would restrict the use of the hormone for potential clinical immunosuppressive therapy” [30,31].

To further determine the therapeutic effect of 1,25(OH)_2_D supplementation for EAE, Cantorna et al. used three different protocols to study the role of 1,25(OH)_2_D supplementation in EAE [32]. In the first protocol, 1,25(OH)_2_D (daily diet 50 ng in female mice and 200 ng in male mice) was administered the day before EAE induction and was found to completely prevent the onset of paralytic disease. In the second protocol, 1,25(OH)_2_D (an initial intraperitoneal dose of 300 ng followed by a daily diet of 50 ng) was initiated at the first paralytic symptoms and was found to ameliorate paralytic symptoms and histopathology scores. In the third protocol, 1,25(OH)_2_D (the same dose as the second protocol) was administered at the peak of paralytic disease and was found to rapidly attenuate the paralytic disease. Calcium levels were also slightly increased in the 1,25(OH)_2_D-treated mice [32]. In addition, using VDR knockout mice, Meehan et al. showed that VDR was necessary for 1,25(OH)_2_D to suppress EAE. A problem in this study was that the 1,25(OH)_2_D dose used also caused severe hypercalcemia [33]. The aforementioned data suggest that 1,25(OH)_2_D supplementation, through VDR, may not only prevent the induction of EAE but also ameliorate established paralytic disease. However, therapeutically effective 1,25(OH)_2_D supplementation is associated with hypercalcemia, which is still a matter of concern.

In addition to the finding that therapeutically effective 1,25(OH)_2_D supplementation is often associated with hypercalcemia, calcium supplementation was also found to suppress EAE [34]. For this reason, it has been suggested that hypercalcemia may be essential for the 1,25(OH)_2_D-mediated suppression of EAE [35]. However, using low calcium diet, Cantorna et al. showed that 1,25(OH)_2_D supplementation could significantly ameliorate EAE without causing hypercalcemia [34]. In another study, Nashold et al. reported that 1,25(OH)_2_D3 at one initial intraperitoneal dose of 100 ng followed by a daily diet of 50 ng, when administered at the peak of paralytic disease, significantly reversed the disease progression. Importantly, this supplementation protocol did not cause hypercalcemia either [36]. An interpretation of the above-mentioned data is that 1,25(OH)_2_D supplementation, if carefully designed, can suppress EAE without causing hypercalcemia.

### 4.2. Role of Supplementation of Native Vitamin D in the Treatment of EAE

As mentioned above, previous studies suggest that direct systemic supplementation of 1,25(OH)_2_D can readily cause hypercalcemia. Since hypercalcemia can cause serious consequences in humans, finding the optimal 1,25(OH)_2_D dose in humans can be challenging, if not impossible. For this reason, researchers have sought to investigate native vitamin D (i.e., a 1,25[OH]_2_D precursor) in animals because it is much safer than 1,25(OH)_2_D. Spach et al. investigated mice that were fed with dietary native vitamin D at an amount three time higher than commercial laboratory mouse diet for 4 weeks and then induced for EAE. When compared to the mice fed with no vitamin D, the vitamin D-fed mice experienced significantly reduced severity of paralytic disease only in female but not male mice [37]. Interestingly, the authors observed that the vitamin D-supplemented female but not male mice showed significant increase in 1,25(OH)_2_D levels in spinal cords after the EAE induction. In contrast, female and male mice with or without the vitamin D supplementation had comparable serum levels of 1,25(OH)_2_D (hence no hypercalcemia) [37]. The data suggest that the ability to maintain a therapeutic level of 1,25(OH)_2_D in CNS during the active disease is critical for protection from the paralytic disease. In addition, the vitamin D supplementation did not confer the protection against EAE in ovariectomized female mice. Moreover, in male mice, even a hypercalcemia dose of native vitamin D supplementation did not confer the protection against EAE. Therefore, the data suggest that female hormone is important for the maintenance of a locally high 1,25(OH)_2_D levels in the CNS during the active disease [37]. In a follow-up study from the above-mentioned group [38], Nashold et al. showed that low-level repletion of 17β-estradiol (E2), which did not prevent EAE induction, restored the vitamin D-mediated resistance to EAE in ovariectomized female mice but did not have any effects in male mice. Interestingly, their data demonstrated that ovariectomized female mice, following the vitamin D supplementation, showed comparable spinal cord 1,25(OH)_2_D concentrations independent of the E2 repletion. This data suggests that the sole increase of spinal cord 1,25(OH)_2_D concentrations is not sufficient to confer protection against EAE. The authors also observed that the E2 repletion significantly enhanced the VDR expression in inflamed CNS only in the presence of the vitamin D supplementation. Considering this fact, the authors speculated that E2 synergized with 1,25(OH)_2_D in inflamed CNS to suppress EAE by enhancing 1,25(OH)_2_D-mediated enhancement of VDR expression [38]. The above findings strongly suggest that gender difference should be considered while vitamin D supplementation is evaluated for the treatment of MS.

In another study, Adzemovic et al. reported that juvenile/adolescent rats supplemented with regular native vitamin D diet for 4 to 5 weeks before EAE induction and those supplemented with vitamin D-deprived diet displayed comparable severity of paralytic disease [39]. Whereas juvenile/adolescent rats supplemented with native vitamin D at an amount fivefold higher than the regular vitamin D dose, when compared with those supplemented with regular vitamin D diet or vitamin D-deprived diet, displayed significant reduction in the severity of paralytic disease following EAE induction. Interestingly, the protective effect of vitamin D at the amount fivefold higher than the regular vitamin D diet was not observed in adult rats. These data may suggest that vitamin D doses higher than those in regular diet is required for disease suppression. In addition, the data also suggest that high dose vitamin D supplementation at adult stage may not be effective in suppressing the severity of paralytic disease. However, in the above study, there was a shortcoming in the experimental design pertaining to the timing of EAE induction. In the rats that received vitamin D supplementation at juvenile/adolescent stage, EAE was induced at 8 weeks of age. Whereas in the rats that received the vitamin D supplementation at adult age, EAE was induced at a much older age (20 weeks) [39]. Relevance of the above findings with respect to humans needs to be explored further, as most of MS patients are adults.

It is important to mention that, although supplementation of native vitamin D is safer than that of 1,25(OH)_2_D, supplementation of such native vitamin D at extremely high doses may still cause hypercalcemia that can result in serious consequences. In a recent study, Hausler et al. showed that mice supplemented with a standard dose of native vitamin D (i.e., 1500 IU/kg food), when compared with those supplemented with low dose of native vitamin D (i.e., <5 IU/kg food), showed comparable severity of paralytic disease. However, when the vitamin D dose increased by 50 folds (i.e., 75,000 IU/kg food), the mice displayed significantly increased severity of paralytic disease, which was accountable to hypercalcemia [40].

## 5. Role of Vitamin D Supplementation in MS

### 5.1. Effects of Supplementation of Native Vitamin D on Disease Activity in MS Patients

Based on previous animal studies, 1,25(OH)_2_D supplementation can readily cause hypercalcemia, whereas supplementation of native vitamin D is relatively safe. Therefore, while assessing native vitamin D supplementation in MS animal models, investigators also studied supplementation of native vitamin D in MS patients. In as early as 1986, such vitamin D supplementation, in combination with calcium and magnesium, was initially evaluated for the treatment of MS [41]. Because of the findings that 1,25(OH)_2_D has at least three biological functions that are potentially beneficial to MS patients, recent clinical investigations began to focus on randomized, placebo-controlled studies to assess the role of supplementation of native vitamin D in the treatment of MS. In one study, using the data from a 96-week randomized and placebo-controlled trial that was initially designed to assess the effect of oral vitamin D3 supplementation (20,000 IU/week) on bone mineral density in MS patients, Kampman et al. reported a modified analysis. This modified post hoc analysis included 35 patients in the vitamin D3 group and 33 in the placebo group. The goal of this analysis was to evaluate potential therapeutic effect of the vitamin D3 supplementation on MS [42]. The results showed that there was no significant difference between groups in annualized relapse rate, expanded disability status scale, MS functional composite components, grip strength, or fatigue [42]. In another study, Soilu-Hanninen et al. performed a one-year study of vitamin D3 supplementation as an add-on treatment to IFN-β1b in MS patients [43]. The study compared 34 MS patients who received an oral supplementation of vitamin D3 (20,000 IU once a week) with 32 MS patients who received a placebo. Their data showed that the vitamin D supplementation reduced the numbers of T2 lesions (*p* = 0.286) and T1 enhancing lesions (*p* = 0.004). In addition, the vitamin D supplementation reduced disability accumulation (*p* = 0.071) and improved timed tandem walk (*p* = 0.076). However, there were no significant differences in adverse events and annual relapse rate. The authors concluded that vitamin D3 supplementation, as an add-on treatment to IFN-β1b, reduced magnetic resonance imaging (MRI) disease activity in MS patients [43]. In the third study, Stein et al. performed a six-month clinical trial [44]. This trial included 11 MS patients who received an oral supplementation of vitamin D2 (an initial dose of 6000 IU twice daily and subsequently adjusted to maintain 25[OH]D levels at 130-175 nM) and 12 MS patients who received a placebo. In addition, all patients received 1000 IU vitamin D daily. Their data showed that no significant treatment differences were detected in the primary MRI endpoints [44]. In the fourth study, Mosayebi et al. performed a six-month trial in which 26 MS patients received 300,000 IU/month of vitamin D3 via intramuscular injection and 33 MS patients received a placebo. This trial showed no significant treatment differences in terms of expanded disability status scale and number of gadolinium-enhancing lesions [45]. Hence, these earlier randomized, placebo-controlled clinical trials do not provide reproducible data in support of the use of vitamin D supplementation for the treatment of MS.

The above-described earlier randomized, placebo-controlled trials may have several drawbacks such as relatively small sample size, insufficient dose, or short length. In this regard, recently published clinical trials tried to overcome these insufficiencies. For instance, Camu et al. studied 63 relapse-remitting MS (RRMS) patients who received an oral vitamin D supplementation (100,000 IU every other week) for 96 weeks and 66 RRMS patients who received a placebo (CHOLINE) [46]. This intervention did not meet the primary outcome, i.e., mean annualized relapse rate. However, for the patients who completed the 2-year follow-up (45 with vitamin D and 45 with placebo), the vitamin D treatment led to significant reductions in annualized relapse rate (*p* = 0.012), new hypointense T1-weighted lesions (*p* = 0.025), volume of hypointense T1-weighted lesions (*p* = 0.031), and progression of expanded disability status scale (*p* = 0.026) [46]. In a multicenter randomized controlled clinical trial (EVIDIMS) [47], Dorr et al. compared high dose vitamin D supplementation (20,400 IU, every other day) with those of low dose vitamin D (400 IU, every other day) as an add-on treatment to IFN-β1b in patients with RRMS or clinically isolated syndrome. Fifty-three patients were randomized (28 in high dose group and 25 in low dose group) and 41 patients completed the 18-month study. The data showed that there were no differences between the two groups regarding relapse rates, disability progression, T2-weighted lesion development, contrast-enhancing lesion development, and brain atrophy [47]. In another study (SOLAR) [48], Hupperts et al. investigated an oral supplementation of high dose vitamin D as an add-on treatment to IFN-β1a in RRMS patients for 48 weeks. This study randomized 229 patients in which 113 patients received a daily supplementation of 14,007 IU vitamin D and 116 patients received a placebo. The data showed that there were no significant differences between the two groups in “no evidence of disease activity” (primary outcome). However, patients in the vitamin D group had better MRI outcomes for combined unique active lesions (*p* = 0.0045) and change from baseline in total volume of T2 lesions (*p* = 0.035) [48]. Notwithstanding the MRI results, data from previous comprehensive randomized, placebo-controlled clinical trials failed to show positive effects on the symptomatology of MS, which is the required endpoint for a successful therapy.

### 5.2. Role of Supplementation of Native Vitamin D in Potentially Pathogenic T Cells in MS Patients

While being evaluated for its potential role in MS disease activity, vitamin D supplementation has also been investigated for its effects on the control of potentially pathogenic T cells. Since IL-17 ^+^ CD4^+^ T cells have been shown to be the major pathogenic cell subset in EAE, IL-17 has been intensively studied in MS patients. In a study of 94 RRMS patients in which 47 patients received a supplementation of 50,000 IU vitamin D3 every five days for 12 weeks and 47 patients received a placebo, Toghianifar et al. reported that the vitamin D3 supplementation appeared to abrogate the non-significant increase of serum IL-17 levels observed in the placebo group [49]. Similar results were seen in another study reported by Golan et al. in which 21 MS patients received 800 IU/day of vitamin D3 (low dose) and 24 MS patients received 4370 IU/day (high dose). At 3 months after the intervention, a significant increase in serum IL-17 levels was observed in the low dose group, which was not seen in the high dose group [50]. In addition, this study did not observe a significant difference in the serum levels of IFN-γ between the low dose and the high dose vitamin D groups. In this respect, Th1 cells, which secrete IFN-γ, are also an important pathogenic T cell subset in MS patients [2]. In contrast, in a study in which 30 RRMS patients received 20,000 IU/week of vitamin D supplementation and 29 received a placebo for 12 months. Aivo et al. reported that there was an increase in the serum IL-17A levels compared to baseline in the vitamin D group (*p* = 0.0666) while the serum IL-17 levels remain similar in the placebo group (*p* = 0.5243) [51]. In another study, 19 MS patients received 10,400 IU/day of vitamin D supplementation (high dose) and 21 received 800 IU/day (low dose) for 6 months [52]. From this study, Sotirchos et al. reported that the high dose vitamin D supplementation significantly reduced the percentages of IL-17^+^CD4^+^ T cells (*p* = 0.016), CD161^+^CD4^+^ T cells (*p* = 0.03), and effector memory CD4^+^ T cells (*p* = 0.021), but increased the percentages of central memory CD4^+^ T cells (*p* = 0.018) and naïve CD4^+^ T cells (*p* = 0.04). These effects were not seen in the low-dose group [52]. In the SOLAR trial in which 30 RRMS patients received 7000 IU/day of vitamin D for 4 weeks followed by 14,000 IU/day up to week 48 and 23 received a placebo, Muris et al. reported no differences in the percentages of IL-17^+^CD4^+^ T cells between the vitamin D3 (*p* = 0.59) and placebo (*p* = 0.96) groups [53]. In an earlier study in which 15 RRMS patient were supplemented with 20,000 IU/day vitamin D3 for 12 weeks [54], Smolders et al. did not observe significant differences in the percentages of CD4^+^IL-17^+^ and CD4^+^IFN-γ^+^ cells before and after the treatment. These results, while not dramatic, are sufficiently positive to justify further exploration into the effects of vitamin D supplementation on potentially pathogenic T cells at local levels such as in the immune system and CNS of MS patients.

### 5.3. Effects of Supplementation of Native Vitamin D on Potentially Immune Regulatory Mechanisms in MS Patients

Treg cells and their associated cytokines (e.g., TGF-β and IL-10) are critical in the control of MS [6,55,56,57]. In one study, mentioned above, Golan et al. showed that there were no differences in the serum levels of IL-10 between the high dose (4370 IU/day) and the low dose (800 IU/day) vitamin D groups [50]. In another study, mentioned above, Avio et al. reported a significant increase in the serum levels of latency activated peptide of TGF-β (*p* = 0.0249) in MS patients who were treated with vitamin D but not those who were treated with placebo (TGF-β is a cytokine used by Treg cells to execute immune regulatory functions) [51]. In addition, this study also showed a mild increase in serum IL-10 levels in the vitamin D-treated patients, which was however not significant (*p* = 0.1466). In contrast, serum IL-10 levels were non-significantly decreased in the placebo-treated patients (*p* = 0.2503) [51]. In the SOLAR trial mentioned above, Muris et al. evaluated the effects of the high vitamin D supplementation on various regulatory cell subsets [53]. In both vitamin D and placebo groups, the authors did not see significant increase in the percentages of various regulatory cell subsets including CD4^+^CD25^+^CD127^−^ natural Treg (nTreg), CD4^+^CD25^+^FoxP3^+^ nTreg, CD4^+^CD25^+^CD127^−^FoxP3^+^ nTreg, CD4^+^CD25^+^CD127^−^CD39^+^ nTreg, CD4^+^CD25^+^CD127^−^CD45RA^−^ memory nTreg, CD4^+^IL-10^+^ induced Treg (iTreg), CD19^+^IL-10^+^ regulatory B cells (Breg) [53]. Finally, in the earlier study mentioned above in which 15 RRMS patients were supplemented with 20,000 IU/day vitamin D3 for 12 weeks [54], Smolders et al. reported that the vitamin D supplementation led to a significant increase in the percentage of CD4^+^IL-10^+^ cells. These results are again sufficiently interesting to warrant the pursuit of the effects of vitamin D supplementation on immune cells at local levels, such as in the immune system and CNS of MS patients.

### 5.4. Role of Metabolic Disorders in the Supplementation of Native Vitamin D for the Treatment of MS

An increasing amount of evidence suggests that disordered lipid metabolism in both peripheral tissues and CNS is associated with MS pathogenesis [58,59]. One report showed that serum levels of low-density lipoprotein and total cholesterol inversely correlated with cognitive function of MS patients [60]. Although we did not find any studies that investigated the effects of vitamin D supplementation on lipid metabolism in MS patients, it is worth discussing this type of studies in other settings such as diabetes. We reason that findings of these studies in other settings may shed light on future similar studies in MS patients. One study investigated blood lipid levels in type 2 diabetes patients at baseline, 3 months, and 6 months following vitamin D supplementation at a daily dose of either 4000 IU or 6000 IU. This study reported significant decrease in total cholesterol and triglycerides in the patients who received 6000 IU vitamin D for 6 months [61]. However, when adjusted for the confounders, the observations were not significant anymore. In another study, Ponda et al. performed a randomized, placebo-controlled trial in which 151 vitamin D insufficient adults (defined as serum 25[OH]D levels < 20 ng/mL) received either 50,000 IU of vitamin D3 weekly or a placebo for 8 weeks. Data from this study showed that the vitamin D supplementation did not improve lipid profile [62]. In the third study, Kane et al. performed an eighteen-week randomized, double-blind, placebo-controlled clinical trial among 26 individuals who had insufficient serum 25(OH)D levels (<25 ng/mL). All the individuals, when entering the study, received a daily supplementation of 1000 IU vitamin D. Subsequently, the vitamin D dose was first adjusted at week 6 to 2000 IU/day if serum 25(OH)D levels were not greater than 25 ng/mL for those individuals whose baseline serum 25(OH)D levels were less than 20 ng/mL or if serum 25(OH)D levels were not increased by at least 25% for those individuals whose baseline serum 25(OH)D levels were 21–25 ng/mL. The dose was further adjusted at week 12 if the above-mentioned 25(OH)D levels were still not met. As a result, vitamin D3 was titrated to 1000 IU/day in 15/26 (58%), 2000 IU/day in 10/26, and 3000 IU/day in 1/26 individuals. Data from the above study showed that serum levels of free but not total 25(OH)D levels inversely correlated with serum levels of triglycerides and low density lipoproteins cholesterol (LDLC) [63]. In the fourth study, Schwetz et al. performed a post hoc analysis of a single-center, double-blind, placebo-controlled clinical trial in which two hundred individuals who had arterial hypertension and serum 25(OH)D levels less than 75 nmol/L were randomized to 2800 IU/day of vitamin D or placebo for 8 weeks. Among the two hundred patients, one hundred sixty-three patients (79 in vitamin D group and 84 in placebo group) had lipid data and these individuals were included in the analysis. The analysis showed that the vitamin D supplementation significantly increased total cholesterol, triglycerides, very low-density lipoproteins (VLDL), low density lipoproteins (LDL), high-density lipoprotein (HDL), triglycerides, apolipoprotein B (ApoB), LDL-ApoB, ApoCII, ApoCIII, phospholipids, and ApoE [64]. Considering the different impacts of vitamin D supplementation on lipid metabolism that were observed under different pathological settings, impact of vitamin D supplementation on lipid metabolism in MS patients should be carefully evaluated.

### 5.5. Role of HLA in the Supplementation of Native Vitamin D for the Treatment of MS

In the past, genome-wide association studies (GWAS) have revealed over 200 genetic loci that are firmly associated with MS susceptibility [65]. Among all these association studies, the major histocompatibility (MHC) gene, HLA-DRB1, has been consistently observed across all populations studied [65]. Additional findings suggest that the genetic association is affected by environmental factors [65]. To understand the influence of vitamin D on the genetic association of MS, Ramgopalan et al. analyzed the entire genomic sequence of the HLA-DRB1, HLA-DQA1, and HLA-DQB1 genes as well as 5 Kb upstream from the transcriptional start sites of these genes that contained promoter regions. Their analysis revealed only one potential vitamin D-responsive element (VDRE) that was located in the proximal promoter region immediately 5′ to the transcriptional start site of HLA-DRB1. Further experiments confirmed that the identified VDRE element was functional and that addition of 1,25(OH)_2_D led to a significant increase in the cell surface expression of HLA-DRB1 specifically and only in HLA-DRB1*15-bearing cells [66]. This data provides strong evidence that 1,25(OH)_2_D can directly modify the expression of HLA-DRB1*15 molecule that has linkage to MS [67]. However, the implication of 1,25(OH)_2_D-mediated modification of HLA-DRB1*15 expression requires further investigation.

### 5.6. Role of Microbiota in the Supplementation of Native Vitamin D for the Treatment of MS

The role of microbiota in the pathogenesis and treatment of paralytic disease has been investigated in animals. In one study, the microbiota was modulated through antibiotic treatment in mice that spontaneously developed EAE. The data showed that microbiota modulation before disease onset prevented the disease development. However, microbiota modulation after disease onset did not affect the ongoing disease [68]. In another study, Cignarella et al. reported that animals with intermittent fasting, when compared to those with a normal diet, showed ameliorated paralytic disease following EAE induction [69]. In addition, in the fasting animals, there was an enrichment of Lactobacillaceae, Bacterioidaceae, and Prevotellaceae families in gut microbiota. Subsequently, the author transplanted fecal microbiome from intermittent fasting mice or normal diet mice into recipient mice that were depleted of microbiota. The mice were then induced for EAE. Their data showed that the fecal microbiome from intermittent fasting mice, but not that from normal diet mice, significantly meliorated paralytic disease in the recipient mice [69]. In summary, the above-mentioned data suggest that the alteration of microbiota changes the susceptibility to EAE induction but has minimal effect on ongoing paralytic disease in animals.

In humans, a recent systemic review suggests that there is no significant difference in microbiota diversity between MS patients and normal healthy controls [70]. However, taxonomic differences in microbiota were noticed. These taxonomic differences indicate a potential role of gut bacteria in MS pathogenesis. In addition, the potential impact of vitamin D supplementation on microbiota in humans was also investigated. In one randomized, placebo-controlled study, 26 vitamin D-insufficient (defined as serum 25[OH] levels < 50 nmol/L), overweight or obese (BMI ≥ 25 kg/m^2^) otherwise healthy adults were recruited [71]. Among the 26 adults, fourteen adults received vitamin D (100,000 IU of loading dose followed by 4000 IU/day for 16 weeks) and 12 adults received a placebo. Fecal microbiota at baseline and week 16 were collected for analysis. The analysis did not see significance in microbiome α-diversity between the two groups at baseline and week 16. However, the adults in the vitamin D group had a higher abundance of genus Lachnospira and a lower abundance of genus Blautia. Furthermore, the adults with 25(OH)D > 75 nmol/L, when compared to those with 25(OH)D < 50 nmol/L, had a higher abundance of genus Coprococcus and had a lower abundance of genus Ruminococcus [71]. In another study, in which 20 adults with vitamin D insufficiency (defined as serum 25[OH]D levels < 30 ng/mL) were provided with 600, 4000, or 10,000 IU/day of oral vitamin D3. Stool samples at baseline and week 8 were collected for the analysis of gut microbiota. The data showed that the vitamin D supplementation led to a dose-dependent increase in bacteria associated with amelioration of inflammatory bowel disease activity [72]. In addition to healthy subjects, the impact of vitamin D supplementation (5000 IU/day for 90 days) on microbiota in MS patients was also studied [73]. Data from this study showed a lower abundance of otherwise operational bacterial unit Faecalibacterium in MS patients. The vitamin D-treated MS patients had an increase in the Akkermansia, Faecalibacterium, and Coprococcus genera. The authors hence concluded that vitamin D supplementation was associated with differences or changes in microbiota [73]. In summary, recent studies show that vitamin D supplementation has effects on the composition of microbiota. However, future studies are warranted to understand how the microbiota changes affect the therapeutic outcome of vitamin D supplementation in MS patients.

## 6. Potential Strategies to Circumvent the Hurdles Facing the Clinical Application of Vitamin D in MS Patients

Previous clinical trials of supplementation with native vitamin D have so far failed to produce the expected outcomes. One of the major barriers is hypercalcemia that can be caused by high doses of 1,25(OH)_2_D as well as extreme high doses of native vitamin D. Hence, strategies that can achieve therapeutic 1,25(OH)_2_D levels without concomitantly causing hypercalcemia are needed.

### 6.1. Role of Low Calcemic 1,25(OH)_2_D Analogues in EAE

One potential strategy to achieve the therapeutic levels of 1,25(OH)_2_D without hypercalcemia is to find a 1,25(OH)_2_D analogue that has the desired 1,25(OH)_2_D function but does not cause hypercalcemia. So far, many 1,25(OH)_2_D low calcemic analogues have been described [74,75]. However, very few of these low calcemic analogues have been studied for their potential therapeutic effects on EAE and MS. In 1995, Slatopolsky et al. reported that 19-Nor-1,25(OH)_2_D_2_ (Paricalcitol) effectively suppressed secondary hyperparathyroidism in uremic rats in the absence of hypercalcemia [76]. To evaluate the role of paricalcitol in the prevention of EAE induction, Zorzella-Pezavento et al. showed that epicutaneous administration of either 1,25(OH)_2_D or paricalcitol at 0.1 μg every other day beginning on day 1 after EAE induction, combined with epicutaneous administration of MOG_35-55_ (150 μg) on day 3 and day 11, effectively ameliorated EAE [77]. Although the 1,25(OH)_2_D administration significantly increased serum calcium levels, the paricalcitol administration only moderately (not significantly) increased serum calcium levels. However, an issue in this study was that the epicutaneous administration of MOG_35-55_ (150 μg) alone was not included in this study. The inclusion of MOG_35-55_ alone in this experimental setting is important for distinguishing the role of MOG_35-55_ administration from the 1,25(OH)_2_D administration. The reason is that the same group reported previously that the MOG_35-55_ administration alone via intraperitoneal route significantly ameliorated EAE, which could not be distinguished from the combined administration of MOG_35-55_ and 1,25(OH)_2_D [78]. Therefore, whether Paricalcitol alone can prevent the EAE induction requires further investigation.

In 2005, Daniel et al. reported another 1,25(OH)_2_D analogue, i.e., 22-ene-25-oxa-vitamin D (ZK156979). They showed that ZK156979 within the range from 10^−5^ to 10^−10^ mol/L dose-dependently suppressed the secretion of IFN-γ, TNF-α, and IL-1β, but dose-dependently enhanced the secretion of IL-4 and IL-10 in peripheral blood mononuclear cells stimulated by phytohemagglutinin [79]. This same group later reported that the ZK156979 at the range from 0.1–2.0 μg/kg did not cause hypercalcemia. Whereas 1,25(OH)_2_D at 0.2 μg/kg caused hypercalcemia. In addition, the ZK156979, administered at 2.0 μg/kg 2 h before or 3 days after the induction of acute colitis, significantly ameliorated experimental colitis by preventing body weight loss, reducing clinical scores, decreasing colon weight, increasing colon length, and mitigating macroscopic and histological scores. In addition, the ZK156979 treatment also decreased the expressions of IFN-γ, TNF-α, and myeloperoxidase (MPO) and T-box transcription factor (T-bet) but increased the expressions of IL-4 and IL-10 in colon tissues [80]. However, it is unknown whether the ZK156979 has a therapeutic effect on EAE without causing hypercalcemia.

The mechanisms by which 1,25(OH)_2_D analogues selectively spare hypercalcemia are not completely understood. It was proposed that the low calcemic mechanism of 22-oxa-1,25(OH)_2_D_3_ (OCT), one 1,25(OH)_2_D analogue, is due to its low affinity for human vitamin D binding protein (DBP). Because of such a low affinity for DBP, OCT, when compared to 1,25(OH)_2_D, has a shorter half-life (2.5 ± 0.3 h versus 7.0 ± 0.6 h), a higher proportion of free form in serum, and more rapid clearance from circulation [74]. It was proposed that, due to such a rapid clearance rate, the OCT was unable to increase intestinal calcium transport and bone mineral mobilization. Whereas the Paricalcitriol has a binding affinity for DBP and circulating half-life that are the same as 1,25(OH)_2_D [81]. The mechanisms underlying the low calcemic property of Paricalcitol are not known. In summary, although many 1,25(OH)_2_D analogues have been described, the mechanisms by which these analogues spare hypercalcemia and still maintain other biological functions are not fully understood. It is possible that the biological functions other than calcium and phosphorous metabolisms of these analogues may not be the same as 1,25(OH)_2_D, which can be the reason that these analogues have not been proven to be effective for the treatment of EAE and MS.

### 6.2. Targeted Delivery of Therapeutic 1,25(OH)_2_D Doses Specifically to a Tissue to Circumvent Hypercalcemia

In contrast, our laboratory has approached the hypercalcemia issue based on the previous report that 1,25(OH)_2_D levels in CNS but not those in serum inversely correlated with disease severity in animals induced for EAE [37]. These data suggest that sufficient 1,25(OH)_2_D levels in a tissue but not those in serum are critical for protecting the tissue from immune-mediated inflammation. For this reason, our group has developed several strategies that are able to target therapeutic 1,25(OH)_2_D doses specifically to a tissue for the treatment of tissue-specific immune-mediated diseases without causing hypercalcemia [6,82,83]. With respect to the treatment of MS, we engineered dendritic cells (DCs) to overexpress 1α-hydroxylase and pulsed such engineered DCs with a myelin antigen [6]. Such myelin antigen-pulsed 1α-hydroxylase-overexpressing DCs were then intravenously administered into animals that were induced for EAE. We showed that such 1α-hydroxylase-overexpressing DCs, when administered at the first sign of paralytic symptoms, ameliorated the progression of paralytic disease without causing hypercalcemia. In addition, such 1α-hydroxylase-overexpressing DCs augmented the induction of CD3^+^Foxp3^+^ T cells in draining lymph nodes. Furthermore, such 1α-hydroxylase-overexpressing DCs did not show any therapeutic effects in mice that were depleted of FoxP3^+^ Treg cells before EAE induction. The data suggest that the 1α-hydroxylase-overexpressing DCs suppress EAE by augmenting the induction of Foxp3^+^ Treg cells in vivo [6]. In summary, our tissue-specific targeting of therapeutic 1,25(OH)_2_D doses can potentially be a novel therapy for MS.

With respect to the potential mechanisms underlying the 1α-hydroxylase-overexpressing DCs, firstly, the 1α-hydroxylase is the physiological enzyme that converts 25(OH)D into 1,25(OH)_2_D [84]. Hence, overexpression of the 1α-hydroxylase in DCs will enhance the DCs’s ability to de novo convert 25(OH)D into 1,25(OH)_2_D. Because it has been well established that DCs home specifically into lymphoid tissues [85,86,87,88], we reason that such 1α-hydroxylase-overexpressing DCs will home specifically into lymphoid tissue. Inside the lymphoid tissues, such 1α-hydroxylase-overexpressing DCs can de novo synthesize locally high concentrations of 1,25(OH)_2_D from 25(OH)D. Such a local de novo synthesis is essential because it will reduce the possibility of increasing systemic 1,25(OH)_2_D levels, thereby circumventing hypercalcemia. In addition, 25(OH)D is the major vitamin D form in blood and as discussed above its levels can be easily raised through supplementation of native vitamin D to provide sufficient substrate for the overexpressed 1α-hydroxylase.

Secondly, we reason that the de novo synthesized local 1,25(OH)_2_D can act on T cells through paracrine action and can also act on the engineered DCs and other DCs through intracrine/autocrine and paracrine actions. In this respect, it has been shown that 1,25(OH)_2_D acts on T cells to augment their expressions of regulatory molecules such as Foxp3 [7,89] and IL-10 [90]. In addition, 1,25(OH)_2_D has also been shown to tolerize DCs [91,92]. Based on the above two findings, it is conceivable that such 1α-hydroxylase-overexpressing DCs may act on T cells to augment the induction of Treg cells and may also augment the induction of Treg cells through tolerizing DCs. Moreover, we reason that the de novo synthesized 1,25(OH)_2_D achieves the above effects through VDR. To support this reasoning, it has been shown that 1,25(OH)_2_D augments the expression of Foxp3 by binding to VDRE in Foxp3 gene [7]. In addition, 1,25(OH)_2_D relies on VDR to induce tolerogenicity in human DCs because such tolerogenicity cannot be induced in DCs from VDR^-/-^ mice [92]. With respect to the role of VDR in the 1,25(OH)_2_D-augmented IL-10 secretion, it has been shown that L-10 gene contains VDRE [93] and that, in macrophages, knockdown of VDR by siRNA abrogates the 1,25(OH)_2_D-augmented IL-10 secretion [94].

Thirdly, if the 1α-hydroxylase-overexpressing DCs do not carry a specific antigen, the specificity of the newly induced Treg cells are diverse and are not specific for demyelinating CNS. To increase the specificity of the newly induced Treg cells for demyelinating CNS, we propose to pulse such 1α-hydroxylase-overexpressing DCs with myelin antigens that are released in demyelinating CNS of MS patients. The rationale is that the myelin antigen-pulsed 1α-hydroxylase-overexpressing DCs will act specifically on T cells with T cell receptors that bind to myelin antigens (i.e., myelin-specific T cells). As a result, the 1α-hydroxylase-overexpressing DCs will induce mostly myelin specific Treg cells that can then specifically target demyelinating CNS in MS patients. We reason that, following in vivo administration, such myelin-specific Treg cells, similar to other immune cells, can travel to CNS during active MS because of the ongoing local inflammation. However, different from the immune cells that are not specific for myelin antigens, myelin-specific Treg cells can recognize and suppress the DCs that present myelin antigens. Because in demyelinating CNS, the myelin antigen-presenting DCs are required for activating myelin-specific pathogenic T cells that are responsible for the ongoing demyelinating inflammation, the suppression of the myelin antigen-presenting DCs blocks the activation of the myelin-specific pathogenic T cells and hence stops the progression of demyelinating inflammation.

Based on the above discussion, one may argue that the function of myelin-specific Treg cells can also be augmented through Foxp3 gene delivery in targets. However, the 1α-hydroxylase-overexpressing DCs have at least three advantages. Firstly, the augmented induction of Foxp3^+^ Treg cells by the 1α-hydroxylase-overexpressing DCs occurs in vivo. In this respect, direct overexpression of Foxp3 may be achieved in T cells through in vitro manipulation. However, in vitro generated Foxp3^+^ Treg cells, following in vivo administration, still face the challenge of potential conversion into pathogenic T cells (i.e., instability of in vitro generated Foxp3^+^ Treg cells) [95,96,97]. Secondly, to avoid generalized immune suppression, Treg cells need to be specific for demyelinating CNS. In this respect, the 1α-hydroxylase-overexpressing DCs can achieve this goal by in vivo augmenting the induction of myelin-specific Treg cells in one step and is easy to implement. If the direct overexpression of Foxp3 in target cells is used, it is necessary to generate myelin-specific T cells in vitro first. Subsequently, the myelin-specific T cells are overexpressed for Foxp3 via gene delivery. Therefore, the direct overexpression of Foxp3 in target cells is time consuming. Thirdly, because 1,25(OH)_2_D also enhances IL-10 expression, the 1α-hydroxylase-overexpressing DCs augment the induction of not only Foxp3^+^ Treg cells but also IL-10^+^ Treg cells. Whereas, direct overexpression of Foxp3 will only enhance Foxp3 expression in target cells.

## 7. Future Directions

Despite confounding issues of hypercalcemia, data from preclinical studies have clearly demonstrated that supplementation of 1,25(OH)_2_D, if carefully designed, significantly ameliorates EAE without causing hypercalcemia. However, finding the optimal 1,25(OH)_2_D doses in humans if not impossible can be challenging. In the past, supplementation of native vitamin D, because of its relatively safe profile, were intensively investigated in animals and humans. In MS animal models, supplementation of native vitamin D reveals several confounding issues such as differential responses based on gender and age. In addition, studies in animal models also revealed other potential factors that may affect the efficacy of vitamin D supplementation for MS treatment such as metabolic disorders, microbiota disorders, and genetic risk alleles. In human MS patients, supplementation of native vitamin D has so far generated mixed outcomes. Therefore, strategies that can achieve therapeutic 1,25(OH)_2_D doses without concomitantly causing hypercalcemia are warranted.

To circumvent the hypercalcemia problem, two potential such strategies have been so far proposed: one is low calcemic 1,25(OH)_2_D analogues and the other is tissue-specific targeting of therapeutic 1,25(OH)_2_D doses. Low calcemic 1,25(OH)_2_D analogues have been intensively investigated for the treatment of other diseases such as secondary hyperparathyroidism. Due to unknown mechanisms underlying the low calcemic property, 1,25(OH)_2_D analogues may also have other biological functions that are different from 1,25(OH)_2_D. It is, therefore, not surprising that there is so far no definitive data to show that these 1,25(OH)_2_D analogues work for EAE and MS. Hence, the role of 1,25(OH)_2_D analogues in EAE and MS requires further investigation. In contrast, studies in EAE have clearly demonstrated that the tissue-specific targeting of therapeutic 1,25(OH)_2_D doses is very promising. Therefore, the tissue-specific targeting of therapeutic 1,25(OH)_2_D doses should be considered for further development into a potential novel therapy for MS. In addition, the confounding issues mentioned above, i.e., metabolic disorders, microbiota disorders, and genetic risk alleles, should also be considered in the future studies of the tissue-specific targeting of therapeutic 1,25(OH)_2_D.

## 8. Conclusions

1,25(OH)_2_D, if carefully dosed, significantly ameliorates EAE without causing hypercalcemia. However, finding the optimal 1,25(OH)_2_D doses in humans is an approach that does not have a satisfactory benefit to risk ratio. Multiple randomized, placebo-controlled clinical trials of supplementation of high dose native vitamin D, although having a safe profile, so far failed to provide reproducible efficacy. Low calcemic 1,25(OH)_2_D analogues have not been definitively shown to be effective for the treatment of EAE and MS. One caveat in the vitamin D supplementation for the treatment of MS is the revelation that 1,25(OH)_2_D levels in CNS but not those in serum inversely correlate with disease activity in animals induced for EAE. This revelation provides a strong rationale for tissue-specific targeting of 1,25(OH)_2_D. In this regard, preclinical studies on tissue-specific targeting of 1,25(OH)_2_D have produced promising results and do so safely. Hence, up until now the tissue-specific targeting appears to be the approach that can take the full advantage of the remarkable immunological and regenerative properties of 1,25(OH)_2_D for the treatment of MS.

## References

[1] Melcon M.O., Correale J., Melcon C.M. (2014). Is it time for a new global classification of multiple sclerosis?. J. Neurol. Sci..

[2] Ando D.G., Clayton J., Kono D., Urban J.L., Sercarz E.E. (1989). Encephalitogenic T cells in the B10.PL model of experimental allergic encephalomyelitis (EAE) are of the Th-1 lymphokine subtype. Cell Immunol..

[3] Cua D.J., Sherlock J., Chen Y., Murphy C.A., Joyce B., Seymour B., Lucian L., To W., Kwan S., Churakova T. (2003). Interleukin-23 rather than interleukin-12 is the critical cytokine for autoimmune inflammation of the brain. Nature.

[4] Lemire J.M., Archer D.C., Beck L., Spiegelberg H.L. (1995). Immunosuppressive actions of 1,25-dihydroxyvitamin D3: Preferential inhibition of Th1 functions. J. Nutr..

[5] Tang J., Zhou R., Luger D., Zhu W., Silver P.B., Grajewski R.S., Su S.B., Chan C.C., Adorini L., Caspi R.R. (2009). Calcitriol suppresses antiretinal autoimmunity through inhibitory effects on the Th17 effector response. J. Immunol..

[6] Li C.H., Zhang J., Baylink D.J., Wang X., Goparaju N.B., Xu Y., Wasnik S., Cheng Y., Berumen E.C., Qin X. (2017). Dendritic cells, engineered to overexpress 25-hydroxyvitamin D 1alpha-hydroxylase and pulsed with a myelin antigen, provide myelin-specific suppression of ongoing experimental allergic encephalomyelitis. Faseb J..

[7] Kang S.W., Kim S.H., Lee N., Lee W.W., Hwang K.A., Shin M.S., Lee S.H., Kim W.U., Kang I. (2012). 1,25-Dihyroxyvitamin D3 promotes FOXP3 expression via binding to vitamin D response elements in its conserved noncoding sequence region. J. Immunol..

[8] Spach K.M., Nashold F.E., Dittel B.N., Hayes C.E. (2006). IL-10 signaling is essential for 1,25-dihydroxyvitamin D3-mediated inhibition of experimental autoimmune encephalomyelitis. J. Immunol..

[9] Hagemeier K., Bruck W., Kuhlmann T. (2012). Multiple sclerosis - remyelination failure as a cause of disease progression. Histol. Histopathol..

[10] De la Fuente A.G., Errea O., van Wijngaarden P., Gonzalez G.A., Kerninon C., Jarjour A.A., Lewis H.J., Jones C.A., Nait-Oumesmar B., Zhao C. (2015). Vitamin D receptor-retinoid X receptor heterodimer signaling regulates oligodendrocyte progenitor cell differentiation. J. Cell Biol..

[11] Kuhlmann T., Miron V., Cui Q., Wegner C., Antel J., Bruck W. (2008). Differentiation block of oligodendroglial progenitor cells as a cause for remyelination failure in chronic multiple sclerosis. Brain.

[12] Fancy S.P., Kotter M.R., Harrington E.P., Huang J.K., Zhao C., Rowitch D.H., Franklin R.J. (2010). Overcoming remyelination failure in multiple sclerosis and other myelin disorders. Exp. Neurol..

[13] Gomez-Pinedo U., Cuevas J.A., Benito-Martin M.S., Moreno-Jimenez L., Esteban-Garcia N., Torre-Fuentes L., Matias-Guiu J.A., Pytel V., Montero P., Matias-Guiu J. (2020). Vitamin D increases remyelination by promoting oligodendrocyte lineage differentiation. Brain Behav..

[14] Bikle D.D. (2014). Vitamin D metabolism, mechanism of action, and clinical applications. Chem. Biol..

[15] Luo W., Hershberger P.A., Trump D.L., Johnson C.S. (2013). 24-Hydroxylase in cancer: Impact on vitamin D-based anticancer therapeutics. J. Steroid Biochem. Mol. Biol..

[16] Zhu J.G., Ochalek J.T., Kaufmann M., Jones G., Deluca H.F. (2013). CYP2R1 is a major, but not exclusive, contributor to 25-hydroxyvitamin D production in vivo. Proc. Natl. Acad. Sci. USA.

[17] Fu G.K., Lin D., Zhang M.Y., Bikle D.D., Shackleton C.H., Miller W.L., Portale A.A. (1997). Cloning of human 25-hydroxyvitamin D-1 alpha-hydroxylase and mutations causing vitamin D-dependent rickets type 1. Mol. Endocrinol..

[18] St-Arnaud R., Messerlian S., Moir J.M., Omdahl J.L., Glorieux F.H. (1997). The 25-hydroxyvitamin D 1-alpha-hydroxylase gene maps to the pseudovitamin D-deficiency rickets (PDDR) disease locus. J. Bone Min. Res..

[19] Li C.H., Tang X., Wasnik S., Wang X., Zhang J., Xu Y., Lau K.W., Nguyen H.B., Baylink D.J. (2019). Mechanistic study of the cause of decreased blood 1,25-Dihydroxyvitamin D in sepsis. Bmc Infect. Dis..

[20] Chanakul A., Zhang M.Y., Louw A., Armbrecht H.J., Miller W.L., Portale A.A., Perwad F. (2013). FGF-23 regulates CYP27B1 transcription in the kidney and in extra-renal tissues. PloS ONE.

[21] Murayama A., Takeyama K., Kitanaka S., Kodera Y., Kawaguchi Y., Hosoya T., Kato S. (1999). Positive and negative regulations of the renal 25-hydroxyvitamin D3 1alpha-hydroxylase gene by parathyroid hormone, calcitonin, and 1alpha,25(OH)2D3 in intact animals. Endocrinology.

[22] Turner R.T., Puzas J.E., Forte M.D., Lester G.E., Gray T.K., Howard G.A., Baylink D.J. (1980). In vitro synthesis of 1 alpha,25-dihydroxycholecalciferol and 24,25-dihydroxycholecalciferol by isolated calvarial cells. Proc Natl. Acad. Sci. USA.

[23] Adams J.S., Rafison B., Witzel S., Reyes R.E., Shieh A., Chun R., Zavala K., Hewison M., Liu P.T. (2014). Regulation of the extrarenal CYP27B1-hydroxylase. J. Steroid Biochem. Mol. Biol..

[24] Zehnder D., Bland R., Williams M.C., McNinch R.W., Howie A.J., Stewart P.M., Hewison M. (2001). Extrarenal expression of 25-hydroxyvitamin d(3)-1 alpha-hydroxylase. J. Clin. Endocrinol. Metab..

[25] St-Arnaud R., Dardenne O., Prud’homme J., Hacking S.A., Glorieux F.H. (2003). Conventional and tissue-specific inactivation of the 25-hydroxyvitamin D-1alpha-hydroxylase (CYP27B1). J. Cell Biochem..

[26] Naja R.P., Dardenne O., Arabian A., St Arnaud R. (2009). Chondrocyte-specific modulation of Cyp27b1 expression supports a role for local synthesis of 1,25-dihydroxyvitamin D3 in growth plate development. Endocrinology.

[27] Schlingmann K.P., Kaufmann M., Weber S., Irwin A., Goos C., John U., Misselwitz J., Klaus G., Kuwertz-Broking E., Fehrenbach H. (2011). Mutations in CYP24A1 and idiopathic infantile hypercalcemia. N. Engl. J. Med..

[28] Goldberg P. (1974). Multiple Sclerosis–Vitamin D and Calcium as Environmental Determinants of Prevalence (a Viewpoint). 1. Sunlight, Dietary Factors and Epidemiology. Int. J. Environ. Stud..

[29] Sintzel M.B., Rametta M., Reder A.T. (2018). Vitamin D and Multiple Sclerosis: A Comprehensive Review. Neurol. Ther..

[30] Lemire J.M., Archer D.C. (1991). 1,25-dihydroxyvitamin D3 prevents the in vivo induction of murine experimental autoimmune encephalomyelitis. J. Clin. Investig..

[31] Cantorna M.T., Hayes C.E., DeLuca H.F. (1996). 1,25-Dihydroxyvitamin D3 reversibly blocks the progression of relapsing encephalomyelitis, a model of multiple sclerosis. Proc. Natl. Acad. Sci. USA.

[32] Cantorna M.T., Woodward W.D., Hayes C.E., DeLuca H.F. (1998). 1,25-dihydroxyvitamin D3 is a positive regulator for the two anti-encephalitogenic cytokines TGF-beta 1 and IL-4. J. Immunol..

[33] Meehan T.F., DeLuca H.F. (2002). The vitamin D receptor is necessary for 1 alpha,25-dihydroxyvitamin D-3 to suppress experimental autoimmune encephalomyelitis in mice. Arch. Biochem. Biophys..

[34] Cantorna M.T., Humpal-Winter J., DeLuca H.F. (1999). Dietary calcium is a major factor in 1,25-dihydroxycholecalciferol suppression of experimental autoimmune encephalomyelitis in mice. J. Nutr..

[35] DeLuca H.F., Plum L. (2017). UVB radiation, vitamin D and multiple sclerosis. Photochem. Photobiol. Sci..

[36] Nashold F.E., Miller D.J., Hayes C.E. (2000). 1,25-dihydroxyvitamin D3 treatment decreases macrophage accumulation in the CNS of mice with experimental autoimmune encephalomyelitis. J. Neuroimmunol..

[37] Spach K.M., Hayes C.E. (2005). Vitamin D3 confers protection from autoimmune encephalomyelitis only in female mice. J. Immunol..

[38] Nashold F.E., Spach K.M., Spanier J.A., Hayes C.E. (2009). Estrogen controls vitamin D3-mediated resistance to experimental autoimmune encephalomyelitis by controlling vitamin D3 metabolism and receptor expression. J. Immunol..

[39] Adzemovic M.Z., Zeitelhofer M., Hochmeister S., Gustafsson S.A., Jagodic M. (2013). Efficacy of vitamin D in treating multiple sclerosis-like neuroinflammation depends on developmental stage. Exp. Neurol..

[40] Hausler D., Torke S., Peelen E., Bertsch T., Djukic M., Nau R., Larochelle C., Zamvil S.S., Bruck W., Weber M.S. (2019). High dose vitamin D exacerbates central nervous system autoimmunity by raising T-cell excitatory calcium. Brain.

[41] Goldberg P., Fleming M.C., Picard E.H. (1986). Multiple sclerosis: Decreased relapse rate through dietary supplementation with calcium, magnesium and vitamin D. Med. Hypotheses.

[42] Kampman M.T., Steffensen L.H., Mellgren S.I., Jorgensen L. (2012). Effect of vitamin D3 supplementation on relapses, disease progression, and measures of function in persons with multiple sclerosis: Exploratory outcomes from a double-blind randomised controlled trial. Mult. Scler..

[43] Soilu-Hanninen M., Aivo J., Lindstrom B.M., Elovaara I., Sumelahti M.L., Farkkila M., Tienari P., Atula S., Sarasoja T., Herrala L. (2012). A randomised, double blind, placebo controlled trial with vitamin D3 as an add on treatment to interferon beta-1b in patients with multiple sclerosis. J. Neurol. Neurosurg. Psychiatry.

[44] Stein M.S., Liu Y., Gray O.M., Baker J.E., Kolbe S.C., Ditchfield M.R., Egan G.F., Mitchell P.J., Harrison L.C., Butzkueven H. (2011). A randomized trial of high-dose vitamin D2 in relapsing-remitting multiple sclerosis. Neurology.

[45] Mosayebi G., Ghazavi A., Ghasami K., Jand Y., Kokhaei P. (2011). Therapeutic effect of vitamin D3 in multiple sclerosis patients. Immunol. Investig..

[46] Camu W., Lehert P., Pierrot-Deseilligny C., Hautecoeur P., Besserve A., Jean Deleglise A.S., Payet M., Thouvenot E., Souberbielle J.C. (2019). Cholecalciferol in relapsing-remitting MS: A randomized clinical trial (CHOLINE). Neurol. Neuroimmunol. Neuroinflamm..

[47] Dorr J., Backer-Koduah P., Wernecke K.D., Becker E., Hoffmann F., Faiss J., Brockmeier B., Hoffmann O., Anvari K., Wuerfel J. (2020). High-dose vitamin D supplementation in multiple sclerosis - results from the randomized EVIDIMS (efficacy of vitamin D supplementation in multiple sclerosis) trial. Mult. Scler. J. Exp. Transl. Clin..

[48] Hupperts R., Smolders J., Vieth R., Holmoy T., Marhardt K., Schluep M., Killestein J., Barkhof F., Beelke M., Grimaldi L.M.E. (2019). Randomized trial of daily high-dose vitamin D3 in patients with RRMS receiving subcutaneous interferon beta-1a. Neurology.

[49] Toghianifar N., Ashtari F., Zarkesh-Esfahani S.H., Mansourian M. (2015). Effect of high dose vitamin D intake on interleukin-17 levels in multiple sclerosis: A randomized, double-blind, placebo-controlled clinical trial. J. Neuroimmunol..

[50] Golan D., Halhal B., Glass-Marmor L., Staun-Ram E., Rozenberg O., Lavi I., Dishon S., Barak M., Ish-Shalom S., Miller A. (2013). Vitamin D supplementation for patients with multiple sclerosis treated with interferon-beta: A randomized controlled trial assessing the effect on flu-like symptoms and immunomodulatory properties. BMC Neurol..

[51] Aivo J., Hanninen A., Ilonen J., Soilu-Hanninen M. (2015). Vitamin D3 administration to MS patients leads to increased serum levels of latency activated peptide (LAP) of TGF-beta. J. Neuroimmunol..

[52] Sotirchos E.S., Bhargava P., Eckstein C., Van Haren K., Baynes M., Ntranos A., Gocke A., Steinman L., Mowry E.M., Calabresi P.A. (2016). Safety and immunologic effects of high- vs low-dose cholecalciferol in multiple sclerosis. Neurology.

[53] Muris A.H., Smolders J., Rolf L., Thewissen M., Hupperts R., Damoiseaux J., Group S.S. (2016). Immune regulatory effects of high dose vitamin D3 supplementation in a randomized controlled trial in relapsing remitting multiple sclerosis patients receiving IFNbeta; the SOLARIUM study. J. Neuroimmunol..

[54] Smolders J., Peelen E., Thewissen M., Cohen Tervaert J.W., Menheere P., Hupperts R., Damoiseaux J. (2010). Safety and T cell modulating effects of high dose vitamin D3 supplementation in multiple sclerosis. PloS ONE.

[55] Astier A.L., Hafler D.A. (2007). Abnormal Tr1 differentiation in multiple sclerosis. J. Neuroimmunol..

[56] Wang X., Zhang J., Baylink D.J., Li C.H., Watts D.M., Xu Y., Qin X., Walter M.H., Tang X. (2016). Targeting Non-classical Myelin Epitopes to Treat Experimental Autoimmune Encephalomyelitis. Sci. Rep..

[57] Danikowski K.M., Jayaraman S., Prabhakar B.S. (2017). Regulatory T cells in multiple sclerosis and myasthenia gravis. J. Neuroinflammation.

[58] Marangon D., Boccazzi M., Lecca D., Fumagalli M. (2020). Regulation of Oligodendrocyte Functions: Targeting Lipid Metabolism and Extracellular Matrix for Myelin Repair. J. Clin. Med..

[59] Gafson A.R., Thorne T., McKechnie C.I.J., Jimenez B., Nicholas R., Matthews P.M. (2018). Lipoprotein markers associated with disability from multiple sclerosis. Sci. Rep..

[60] Noori H., Gheini M.R., Rezaeimanesh N., Saeedi R., Rezaei Aliabadi H., Sahraian M.A., Naser Moghadasi A. (2019). The correlation between dyslipidemia and cognitive impairment in multiple sclerosis patients. Mult. Scler. Relat. Disord.

[61] Exebio J.C., Ajabshir S., Campa A., Li T., Zarini G.G., Huffman F.G. (2018). The Effect of Vitamin D Supplementation on Blood Lipids in Minorities with Type 2 Diabetes. Int. J. Diabetes Clin. Res..

[62] Ponda M.P., Dowd K., Finkielstein D., Holt P.R., Breslow J.L. (2012). The short-term effects of vitamin D repletion on cholesterol: A randomized, placebo-controlled trial. Arter. Thromb. Vasc. Biol..

[63] Kane L., Moore K., Lutjohann D., Bikle D., Schwartz J.B. (2013). Vitamin D3 effects on lipids differ in statin and non-statin-treated humans: Superiority of free 25-OH D levels in detecting relationships. J. Clin. Endocrinol. Metab..

[64] Schwetz V., Scharnagl H., Trummer C., Stojakovic T., Pandis M., Grubler M.R., Verheyen N., Gaksch M., Zittermann A., Aberer F. (2018). Vitamin D supplementation and lipoprotein metabolism: A randomized controlled trial. J. Clin. Lipidol..

[65] Canto E., Oksenberg J.R. (2018). Multiple sclerosis genetics. Mult. Scler..

[66] Ramagopalan S.V., Maugeri N.J., Handunnetthi L., Lincoln M.R., Orton S.M., Dyment D.A., Deluca G.C., Herrera B.M., Chao M.J., Sadovnick A.D. (2009). Expression of the multiple sclerosis-associated MHC class II Allele HLA-DRB1*1501 is regulated by vitamin D. Plos Genet..

[67] Handunnetthi L., Ramagopalan S.V., Ebers G.C. (2010). Multiple sclerosis, vitamin D, and HLA-DRB1*15. Neurology.

[68] Godel C., Kunkel B., Kashani A., Lassmann H., Arumugam M., Krishnamoorthy G. (2020). Perturbation of gut microbiota decreases susceptibility but does not modulate ongoing autoimmune neurological disease. J. Neuroinflammation.

[69] Cignarella F., Cantoni C., Ghezzi L., Salter A., Dorsett Y., Chen L., Phillips D., Weinstock G.M., Fontana L., Cross A.H. (2018). Intermittent Fasting Confers Protection in CNS Autoimmunity by Altering the Gut Microbiota. Cell Metab..

[70] Mirza A., Forbes J.D., Zhu F., Bernstein C.N., Van Domselaar G., Graham M., Waubant E., Tremlett H. (2020). The multiple sclerosis gut microbiota: A systematic review. Mult. Scler. Relat. Disord..

[71] Naderpoor N., Mousa A., Fernanda Gomez Arango L., Barrett H.L., Dekker Nitert M., de Courten B. (2019). Effect of Vitamin D Supplementation on Faecal Microbiota: A Randomised Clinical Trial. Nutrients.

[72] Charoenngam N., Shirvani A., Kalajian T.A., Song A., Holick M.F. (2020). The Effect of Various Doses of Oral Vitamin D3 Supplementation on Gut Microbiota in Healthy Adults: A Randomized, Double-blinded, Dose-response Study. Anticancer Res..

[73] Cantarel B.L., Waubant E., Chehoud C., Kuczynski J., DeSantis T.Z., Warrington J., Venkatesan A., Fraser C.M., Mowry E.M. (2015). Gut microbiota in multiple sclerosis: Possible influence of immunomodulators. J. Investig. Med..

[74] Dusso A.S., Negrea L., Gunawardhana S., Lopez-Hilker S., Finch J., Mori T., Nishii Y., Slatopolsky E., Brown A.J. (1991). On the mechanisms for the selective action of vitamin D analogs. Endocrinology.

[75] Brown A.J. (1998). Vitamin D analogues. Am. J. Kidney Dis..

[76] Slatopolsky E., Finch J., Ritter C., Denda M., Morrissey J., Brown A., DeLuca H. (1995). A new analog of calcitriol, 19-nor-1,25-(OH)2D2, suppresses parathyroid hormone secretion in uremic rats in the absence of hypercalcemia. Am. J. Kidney Dis..

[77] Zorzella-Pezavento S.F.G., Mimura L.A.N., Fraga-Silva T.F.C., Ishikawa L.L.W., Franca T.G.D., Sartori A. (2017). Experimental Autoimmune Encephalomyelitis Is Successfully Controlled by Epicutaneous Administration of MOG Plus Vitamin D Analog. Front. Immunol..

[78] Chiuso-Minicucci F., Ishikawa L.L., Mimura L.A., Fraga-Silva T.F., Franca T.G., Zorzella-Pezavento S.F., Marques C., Ikoma M.R., Sartori A. (2015). Treatment with Vitamin D/MOG Association Suppresses Experimental Autoimmune Encephalomyelitis. PloS ONE.

[79] Daniel C., Schlauch T., Zugel U., Steinmeyer A., Radeke H.H., Steinhilber D., Stein J. (2005). 22-ene-25-oxa-vitamin D: A new vitamin D analogue with profound immunosuppressive capacities. Eur. J. Clin. Investig..

[80] Daniel C., Radeke H.H., Sartory N.A., Zahn N., Zuegel U., Steinmeyer A., Stein J. (2006). The new low calcemic vitamin D analog 22-ene-25-oxa-vitamin D prominently ameliorates T helper cell type 1-mediated colitis in mice. J Pharm. Exp..

[81] Takahashi F., Finch J.L., Denda M., Dusso A.S., Brown A.J., Slatopolsky E. (1997). A new analog of 1,25-(OH)2D3, 19-NOR-1,25-(OH)2D2, suppresses serum PTH and parathyroid gland growth in uremic rats without elevation of intestinal vitamin D receptor content. Am. J. Kidney Dis..

[82] Li B., Baylink D.J., Walter M.H., Lau K.H., Meng X., Wang J., Cherkas A., Tang X., Qin X. (2015). Targeted 25-hydroxyvitamin D3 1alpha-hydroxylase adoptive gene therapy ameliorates dss-induced colitis without causing hypercalcemia in mice. Mol. Ther..

[83] Xu Y., Cheng Y., Baylink D.J., Wasnik S., Goel G., Huang M., Cao H., Qin X., Lau K.W., Chan C. (2019). In vivo generation of gut-homing regulatory T cells for the suppression of colitis. J. Immunol..

[84] Gray R.W., Omdahl J.L., Ghazarian J.G., DeLuca H.F. (1972). 25-Hydroxycholecalciferol-1-hydroxylase. Subcellular location and properties. J. Biol. Chem..

[85] Lee H.W., Yoon S.Y., Singh T.D., Choi Y.J., Lee H.J., Park J.Y., Jeong S.Y., Lee S.W., Ha J.H., Ahn B.C. (2015). Tracking of dendritic cell migration into lymph nodes using molecular imaging with sodium iodide symporter and enhanced firefly luciferase genes. Sci. Rep..

[86] MartIn-Fontecha A., Sebastiani S., Hopken U.E., Uguccioni M., Lipp M., Lanzavecchia A., Sallusto F. (2003). Regulation of dendritic cell migration to the draining lymph node: Impact on T lymphocyte traffic and priming. J. Exp. Med..

[87] Creusot R.J., Yaghoubi S.S., Chang P., Chia J., Contag C.H., Gambhir S.S., Fathman C.G. (2009). Lymphoid-tissue-specific homing of bone-marrow-derived dendritic cells. Blood.

[88] Randolph G.J., Angeli V., Swartz M.A. (2005). Dendritic-cell trafficking to lymph nodes through lymphatic vessels. Nat. Rev. Immunol..

[89] Jeffery L.E., Burke F., Mura M., Zheng Y., Qureshi O.S., Hewison M., Walker L.S., Lammas D.A., Raza K., Sansom D.M. (2009). 1,25-Dihydroxyvitamin D3 and IL-2 combine to inhibit T cell production of inflammatory cytokines and promote development of regulatory T cells expressing CTLA-4 and FoxP3. J. Immunol..

[90] Bartels L.E., Jorgensen S.P., Agnholt J., Kelsen J., Hvas C.L., Dahlerup J.F. (2007). 1,25-dihydroxyvitamin D3 and dexamethasone increase interleukin-10 production in CD4+ T cells from patients with Crohn’s disease. Int. Immunopharmacol..

[91] Adorini L., Penna G., Giarratana N., Uskokovic M. (2003). Tolerogenic dendritic cells induced by vitamin D receptor ligands enhance regulatory T cells inhibiting allograft rejection and autoimmune diseases. J. Cell Biochem..

[92] Ferreira G.B., Vanherwegen A.S., Eelen G., Gutierrez A.C.F., Van Lommel L., Marchal K., Verlinden L., Verstuyf A., Nogueira T., Georgiadou M. (2015). Vitamin D3 Induces Tolerance in Human Dendritic Cells by Activation of Intracellular Metabolic Pathways. Cell Rep..

[93] Heine G., Niesner U., Chang H.D., Steinmeyer A., Zugel U., Zuberbier T., Radbruch A., Worm M. (2008). 1,25-dihydroxyvitamin D(3) promotes IL-10 production in human B cells. Eur. J. Immunol..

[94] Zhang X., Zhou M., Guo Y., Song Z., Liu B. (2015). 1,25-Dihydroxyvitamin D(3) Promotes High Glucose-Induced M1 Macrophage Switching to M2 via the VDR-PPARgamma Signaling Pathway. Biomed. Res. Int..

[95] Komatsu N., Okamoto K., Sawa S., Nakashima T., Oh-hora M., Kodama T., Tanaka S., Bluestone J.A., Takayanagi H. (2014). Pathogenic conversion of Foxp3+ T cells into TH17 cells in autoimmune arthritis. Nat. Med..

[96] Joller N., Kuchroo V.K. (2014). Good guys gone bad: exTreg cells promote autoimmune arthritis. Nat. Med..

[97] Zhou X., Bailey-Bucktrout S.L., Jeker L.T., Penaranda C., Martinez-Llordella M., Ashby M., Nakayama M., Rosenthal W., Bluestone J.A. (2009). Instability of the transcription factor Foxp3 leads to the generation of pathogenic memory T cells in vivo. Nat. Immunol..

